# Angled manual traction and usual care for cervical radiculopathy: rationale and a protocol for a pilot randomized controlled trial (pAMTLER)

**DOI:** 10.3389/fmed.2025.1695623

**Published:** 2025-12-19

**Authors:** Shaojun Liao, Junhan Huang, Zhuolong Peng, Yu Hou, Guoyi Su, Yongjin Li, Zehuai Wen, Dingkun Lin

**Affiliations:** 1 Minimally Invasive Spine Surgery Center, Guangdong Provincial Hospital of Chinese Medicine, Guangzhou, China; 2 Research Team on the Prevention and Treatment of Spinal Degenerative Disease, Guangdong Provincial Academy of Chinese Medical Sciences, Guangzhou, China; 3 Guangzhou Key Laboratory of Cervical Spine Biomechanics, Guangzhou, China; 4 Chinese Medicine Guangdong Laboratory, Zhuhai, China; 5 Center for Clinical Research, Guangdong Provincial Hospital of Chinese Medicine, Guangzhou, China

**Keywords:** angled manual traction, cervical radiculopathy, conservative management, pilot study, randomized controlled trial, study protocol

## Abstract

**Background:**

Angled manual traction (AMT) is widely used for relieving moderate-to-severe cervicobrachial pain in patients with cervical radiculopathy (CR), yet its effectiveness has yet to be established by rigorous full randomized controlled trials (RCTs). We have designed an external pilot to evaluate the feasibility of a future large-scale, definitive RCT on AMT for CR.

**Methods:**

48 CR participants with cervicobrachial pain (numeric rating scale ≥ 4) will be enrolled in a five-period pilot RCT and randomly assigned to receive either usual care alone or AMT plus usual care for 4 consecutive periods (each period defined as 7 days). The primary outcome will be feasibility, focusing on enrolment rate, retention rate, and protocol adherence. The secondary outcomes include pain in the cervicobrachial region, upper extremity numbness, muscle weakness, upper extremity and neck function, analgesic consumption (non-steroidal anti-inflammatory drugs and opiates), work ability, quality of life, emotional well-being prior to administering treatment at each period’s initial visit, as well as safety and intervention costs during the trial. We employ linear mixed-effect models on the efficacy-related outcome measures to assess the changes within and between groups over time, and determine the statistical trends of effectiveness.

**Results:**

We expect the trial to be completed by June 2026, with successful pilot targets defined as achieving ≥ 25% enrolment, ≥80% adherence, ≥80% retention, and superior health outcomes in the AMT add-on arm compared with usual care.

**Conclusion:**

This external pilot trial will provide robust data on feasibility and outcome variability for power calculations in the proposed future confirmatory RCT on AMT for CR. This pilot RCT will be invaluable to the design and management of the subsequent full-scale RCT.

**Trial registration:**

Chinese Clinical Trial Registry (ChiCTR): https://www.chictr.org.cn/showproj.html?proj=236348 ChiCTR2400087289.

## 1 Introduction

Cervical radiculopathy (CR) is a term used to describe a consequence triggered by mechanical compression of nerve roots within the neural foramen due to age-related degenerative changes in the cervical spine, i.e., herniated disk material, hypertrophied facet joints, and/or uncovertebral joints (1–5). It is a prevalent, debilitating cervical spine condition characterized by pain in the cervicobrachial region and abnormal neurologic signs along the path of innervation of the affected nerve root, including paraesthesia, numbness, muscle weakness and diminished deep tendon reflexes. The annual incidence has been reported to be between 0.83 and 1.79 per 1,000 person-years, with an estimated prevalence from 1.21 to 5.8 per 1,000 (6). CR is frequently described as a self-limiting disorder with a favorable prognosis (7, 8). However, pain during the first weeks to months is often excruciating (9), and most patients report experienced continuous, fluctuating pain for up to 24–36 months before achieving complete recovery (10, 11). Such pain contributes to impaired physical function, poor mental well-being, limited regular activities, and isolated social life (12–14). It has been reported that approximately 352.0 cases per 100,000 individuals worldwide have suffered from disability resulting from CR over the past three decades (15). Patients with CR are vulnerable to experiencing varying degrees of work ability impairment due to both physiological disturbances and psychological distress (13, 14, 16). Hence, they may be at a higher risk of exclusion from the labor market due to frequent sick leave, work absenteeism, and even failure to execute work duties (13, 14). Patients with CR are also believed to experience poorer economic outcomes as a result of work productivity loss, with an estimated average willingness-to-pay far exceeding $38.0 per month for a 1-point reduction in pain (17).

Relieving pain, improving neurologic function, and preventing recurrence are the primary treatment objectives to facilitate CR patients’ return to their usual activities and work (1, 3, 18). Conservative management is universally considered the first course for CR due to its favorable natural history, where non-steroidal anti-inflammatory drugs (NSAIDs) are typically the primary options, with possible escalation to opioids when pain remains uncontrolled (1, 8, 18, 19). Multimodal care combining oral analgesics with physiotherapy, which includes but is not limited to collar immobilization, traction, and manual therapy, is generally recommended as the initial prescription for CR (8, 18–20). However, the existing conservative strategies are of limited value in accelerating improvements in pain and function (20, 21). The current body of knowledge highlights a critical need for additional scalable, safe pain relief strategies for patients with CR.

Angled manual traction (AMT) is an integrative Chinese-Western protocol originating from clinical observations that forced cervical anteflexion alleviates pain in middle-aged and elderly patients with CR (22). This combined modality sequentially combines two *tuina* techniques—pressing-kneading manipulation and traction manipulation—with mechanical traction. Both are performed with the cervical spine held in anterior flexion at variable angles, which are determined by the pathoanatomic level of nerve-root compression. Over nearly two decades of CR management, AMT has shown excellent alleviation of moderate-to-severe cervicobrachialgia, with benefits presenting immediately post-treatment and extending to functional deficits (23). It has also shown considerable safety (24). Unlike the Kaltenborn method (25, 26), which is based on the concave-convex rule to restore physiological motion in hypomobile joints through graded traction and gliding, AMT appears more consistent with the pathogenesis of CR in middle-aged and elderly individuals. In these patients, neural foramen stenosis is typically caused by degenerative loss of disk height and the resulting compensatory hypertrophy of intervertebral joints to stabilize the cervical spine (27). Our ongoing biomechanics experiments on cervical cadaveric species (28) preliminarily show that the heights of intervertebral foramina at different cervical levels (C4-C5, C5-C6, and C6-C7) tend to increase with greater flexion angles (Appendix S1). During the experiments, the cervical spine specimens were subjected to a 60 N axial tensile force at various angles of anterior cervical flexion. Magnetic resonance imaging in middle-aged and elderly patients with CR (24, 29) also indicated that both the sagittal diameter and the cross-sectional area of the cervical spinal canal following AMT—characterized by applying traction to the cervical spine with the neck positioned in 20°–45° flexion—were greater than those measured when traction was performed in the neutral (0° extension) or retraction (15° extension) positions. Additionally, the upward pulling-stretching force applied along the longitudinal axis of the cervical spine during traction manipulation of AMT might elicit a more pronounced and immediate reduction in intradiscal pressure than mechanical traction alone, thereby achieving rapid pain relief (30). AMT has consequently been regarded as a viable proactive intervention for reducing surgical risks in CR patients. Anecdotally, patients with CR receiving AMT have also been observed to have faster work resumption, indicating that this complex intervention program may lower health care costs, improve economic outcomes, and thus be cost-effective.

Given the insufficiency of current conservative therapies, AMT, with its wide-ranging physical and psychological benefits, is emerging as another promising therapy for patients with CR. Nevertheless, these conclusions are largely derived from anecdotal experiences or observations of poor quality, such as those which lack systematic design and experimental comparators, as well as those with small and poorly represented samples, non-standard measures, insufficient follow-up periods, and/or incomplete reporting. Thus, the definitive role of AMT in CR remains to be established through well-designed randomized controlled trials (RCTs) with substantial sample sizes. However, the available information cannot provide robust data to inform the design and delivery of an RCT with solid methodologies. Given these considerations, we propose a small-scale pilot RCT. We hope the findings of this preliminary trial will provide valuable insight into the feasibility, design, and even management of a future full-scale RCT with adequate power.

## 2 Objectives

The aim of this external pilot study is to ascertain the feasibility of a definitive full-scale RCT that determines whether AMT plus usual care is superior to usual care alone in clinical efficacy and cost-effectiveness regarding cervicobrachialgia reduction. In this study, we will (1) evaluate the recruitment and retention of participants, treatment adherence, and completion of proposed outcome measures to inform the feasibility and acceptability of study design; (2) and estimate the variability of efficacy-related outcomes to preliminarily inform sample size calculation for a future fully powered RCT.

## 3 Materials and methods

### 3.1 Study design

**Figure 1:**
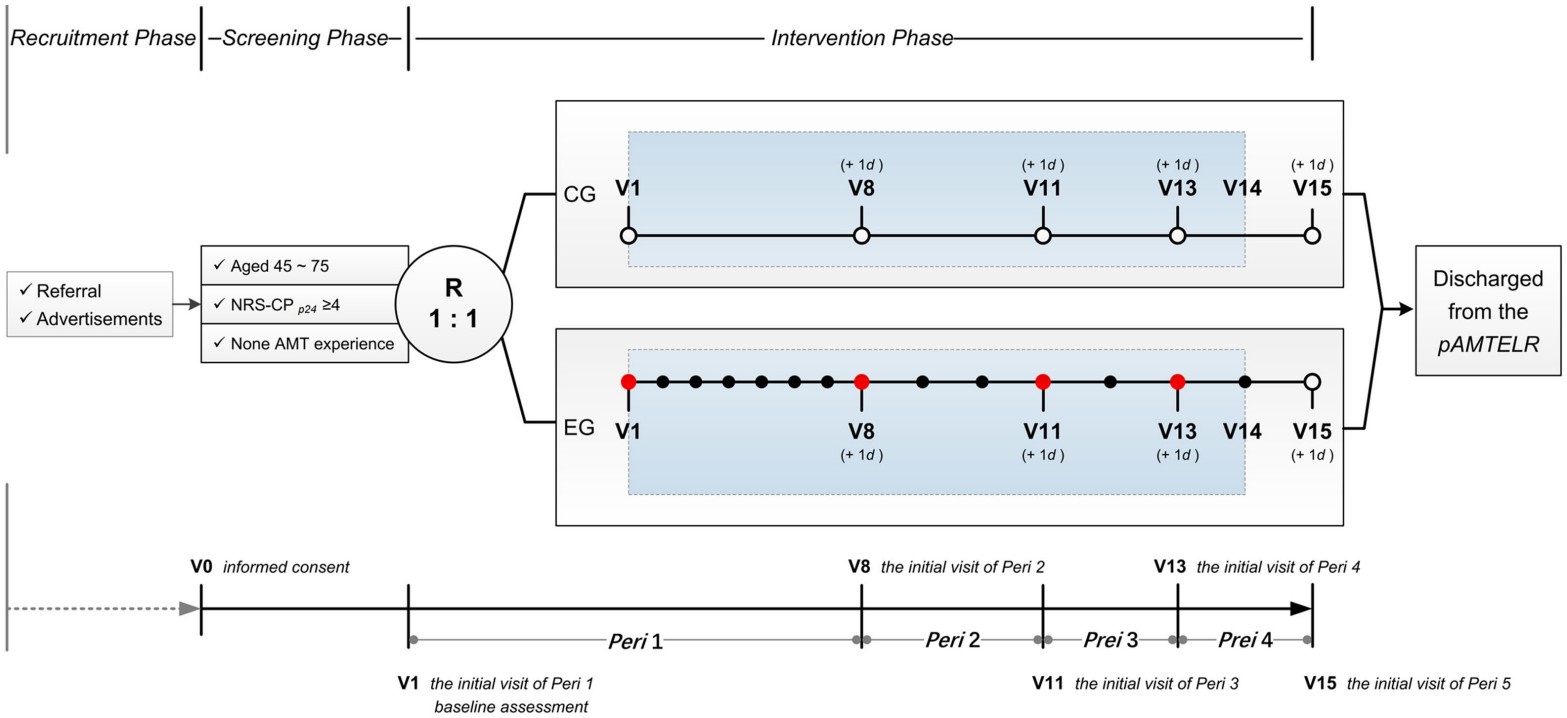
pAMTLER study design. CR, cervical radiculopathy; R, randomized allocation; CG, control group; EG, experimental group; V, visit; Peri, period; NRS-CP_p24_, having cervicobrachialgia over the previous 24 h on NRS; +1 d, data premitted to be collected within 1 day after the scheduled time. 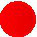 collecting data on efficacy- and cost-related outcome measures, followed by administering AMT; 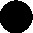, collecting data on cost-related outcome measures, followed by administering AMT; 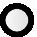, collecting data on efficacy- and cost-related outcome measures; 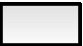, recording adverse events, adverse reactions and concomitant medication during the trial; 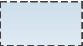, receiving usual care for 4 periods. Baseline assessment including socio-demographics (age, sex, education level, employment status, health insurance, etc.), medical history (comorbidities, concomitant medications, details related to the cervicobrachial pain episode, etc.) and physical examination (dominant hand, abnormal deep tendon reflexes, radiculopathy level shown on magnetic resonance imaging, etc.).

**Figure 2:**
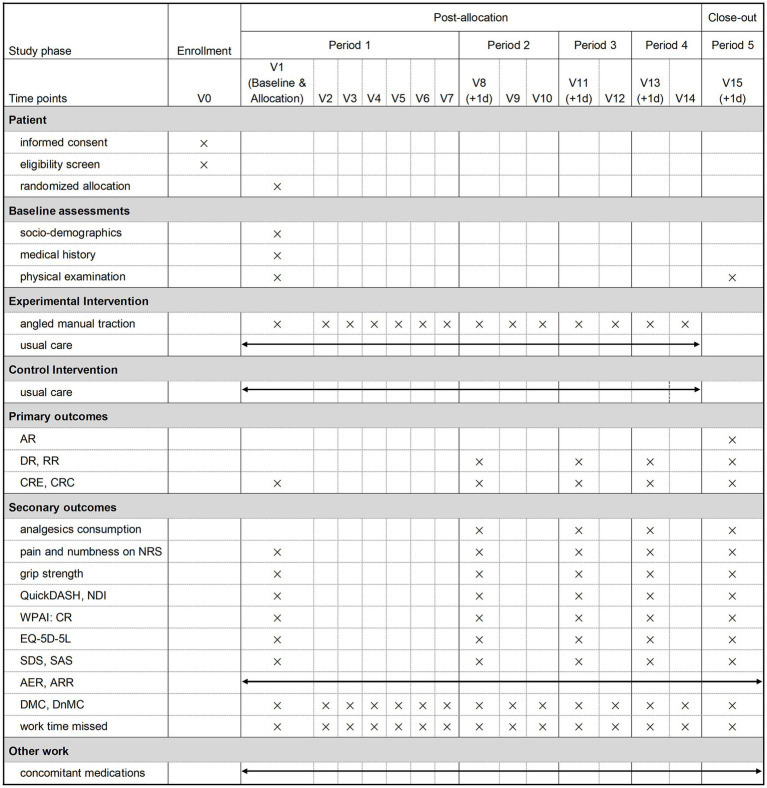
pAMTLER SPIRIT schedule. AR, adherence rate; DR, drop-out rate; RR, retention rate; CRE, completion rates of efficacy-related outcome measures; CRC, completion rates of cost-related outcome measures; NRS, cervicobrachial pain, upper extremity pain and numbness, neck pain, and shoulder pain graded by the numeric rating scale; QuickDASH, quick disabilities of the arm, shoulder, and hand questionnaire; NDI, neck disability index; analgesics consumption, NSAID consumption per period and opiate consumption per period; WPAI-CR, work productivity and activity impairment questionnaire: cervical radiculopathy; EQ-5D-5L, euroqol 5-dimensional descriptive system; SDS, Zung’s self-rating depression scale; SAS, Zung’s self-rating anxiety scale; AER, adverse event rates; ARR, adverse reaction rates; DMC, direct medical costs; DnMC, direct non-medical costs. In baseline assessment, socio-demographics (e.g., age, sex, education level, employment status, health insurance), medical history (e.g., comorbidities, concomitant medications, details related to the cervicobrachial pain episode), physical examination (e.g., dominant hand, abnormal deep tendon reflexes) and radiculopathy level are shown on magnetic resonance imaging.

The **p**ilot study on **A**ngled **M**anual **T**raction and usua**L** car**E** for cervical **R**adiculopathy (pAMTLER) will be an open-label RCT with two parallel groups and blind evaluation. In this five-period (7 days per period) trial with a total of 15 visits, 48 eligible participants with CR will be enrolled. All enrolled participants will receive usual care for four periods. The 24 participants randomly assigned to the experimental group will also receive 14 sessions of AMT. The baseline will be defined as the pre-treatment assessment during the initial visit in the first period after randomization. Participants will be scheduled to complete the outcome assessments prior to treatment during the initial visit in each period. Figure 1 illustrates the study design. The pAMTLER will be conducted at the Guangdong Provincial Hospital of Chinese Medicine (GPHCM), Guangzhou, China, with approval from the Ethics Committee at GPHCM (approval number: BF2024-156-01). The study protocol was registered with ChiCTR2400087289, and is reported according to the Standard Protocol Items: Recommendations for Interventional Trials (SPIRIT) (31) (Appendix S2). Figure 2 overviews the participant flow and details the assessed content at each scheduled visit (Appendix S3).

### 3.2 Study population

Participants having all of the following conditions will be included:

1. Satisfied the following diagnostic criteria of CR, which was drafted according to the *Expert Consensus on Cervical Radiculopathy Standardized Diagnosis and Treatment* issued in China in 2015 (32), the *Diagnosis and Treatment of Cervical Radiculopathy from Degenerative Disorders* released by the North American Spine Society in 2010 (7), and pertinent literature about CR diagnostic accuracy (33):

- suffering from cervicobrachialgia, accompanying numbness, weakness, and abnormal deep tendon reflexes in the upper limb(s).
- having positive provocation tests such as Spurling’s test, distraction test, or brachial plexus tension test.
- pathologic level indicated by symptoms and signs in line with the impinged cervical nerve roots shown on magnetic resonance imaging.

1. Aged ≥ 45 to ≤ 75.
2. cervicobrachialgia over the previous 24 h ≥ 4 on numerical rating scale (NRS)
3. No AMT experience.
4. Signed informed consent.

Participants with any of the following phenomenon will be excluded:

1. Comorbidities that could contribute to cervicobrachialgia (e.g., shoulder bursitis, thoracic outlet syndrome, spinal muscular atrophy, cubital tunnel syndrome, tennis elbow, carpal tunnel syndrome, angina pectoris and pathological changes of cervical vertebrae like tuberculosis, neoplasms).
2. Congenital spine diseases (scoliosis, abnormalities, etc.), cervical surgery history, spinal cord injury history, osteomyelitis, severe osteoporosis or any other types of cervical spondylosis (cervical myelopathy, mixed-type cervical spondylosis, etc.).
3. Contraindications against NSAIDs or opiates (e.g., hypersensitivity to NSAIDs or opiates, active or relapsing peptic ulcer or gastrointestinal bleeding, NSAID-related gastrointestinal bleeding or perforation, gastrointestinal obstruction, hepatic failure, renal failure with glomerular filtration rate <15 mL/min/1.73 m^2^, heart failure with New York Heart Association class IV, bronchial asthma, and on prescribed monoamine oxidase inhibitors within 2 weeks).
4. Active malignancy.
5. Planning surgery during the study period.
6. Failing to understand and/or follow the study protocol due to disabilities (blindness, deafness, dumbness, intellectual disability, psychiatric disorder, etc.).
7. Gravida, lactating women, or individuals planning to conceive during the trial.
8. Participating in other clinical trials within 1 month or planning to participate in other concurrent clinical studies.
9. Any other conditions investigators deem inappropriate.

### 3.3 Recruitment

Potential participants will be recruited by clinicians at GPHCM’s orthopedic outpatient clinics, as well as through advertisements issued via GPHCM’s official accounts, social media platforms including TikTok and WeChat (Tencent), and newspapers. Interested individuals will be encouraged to contact the researcher (a registered research physician responsible for usual care), using the private telephone line listed on the recruitment flyers in clinic or the advertisements. The research physician will be independent of the outcome assessment and random allocation process. He will identify candidates who fulfill the CR diagnostic criteria among the approached individuals, and invite them to participate in the pAMTLER after informing them of the study’s purpose, procedure, as well as participation benefits and potential risks. Willing candidates will be further assessed for eligibility according to the inclusion and exclusion criteria after voluntarily signing written informed consent forms. Those eligible to participate in the post-evaluation will then be consecutively enrolled and randomized. The randomized individuals can withdraw participation consent at any time during the trial without prejudice. Details related to screening, enrolment, withdrawal, and reasons for non-participation will be documented in a prearranged register.

### 3.4 Randomization and blinding

Eligible participants will be assigned to either the experimental group (AMT add-on) or the control group (usual care alone) at a 1:1 ratio with a random allocation schedule, which will be generated by a third-party biostatistician using R software (version 4.3.0). The treatment allocation table will be stratified by baseline cervicobrachial pain level (NRS < 7 or ≥ 7) and sex, then randomized with varying block sizes to avoid insufficient concealment caused by limited sample size. The random allocation schedule will be concealed in sequentially numbered, sealed, opaque envelopes, and managed by the biostatistician. He will reveal the treatment allocation via phone according to the participants’ enrolment sequences.

Due to the nature of the intervention, it will be impossible to blind the participants and treatment providers. However, outcome assessors, data managers and statistical analysts will be unaware of the treatment allocations.

### 3.5 Interventions

#### 3.5.1 Usual care

**Figure 3:**
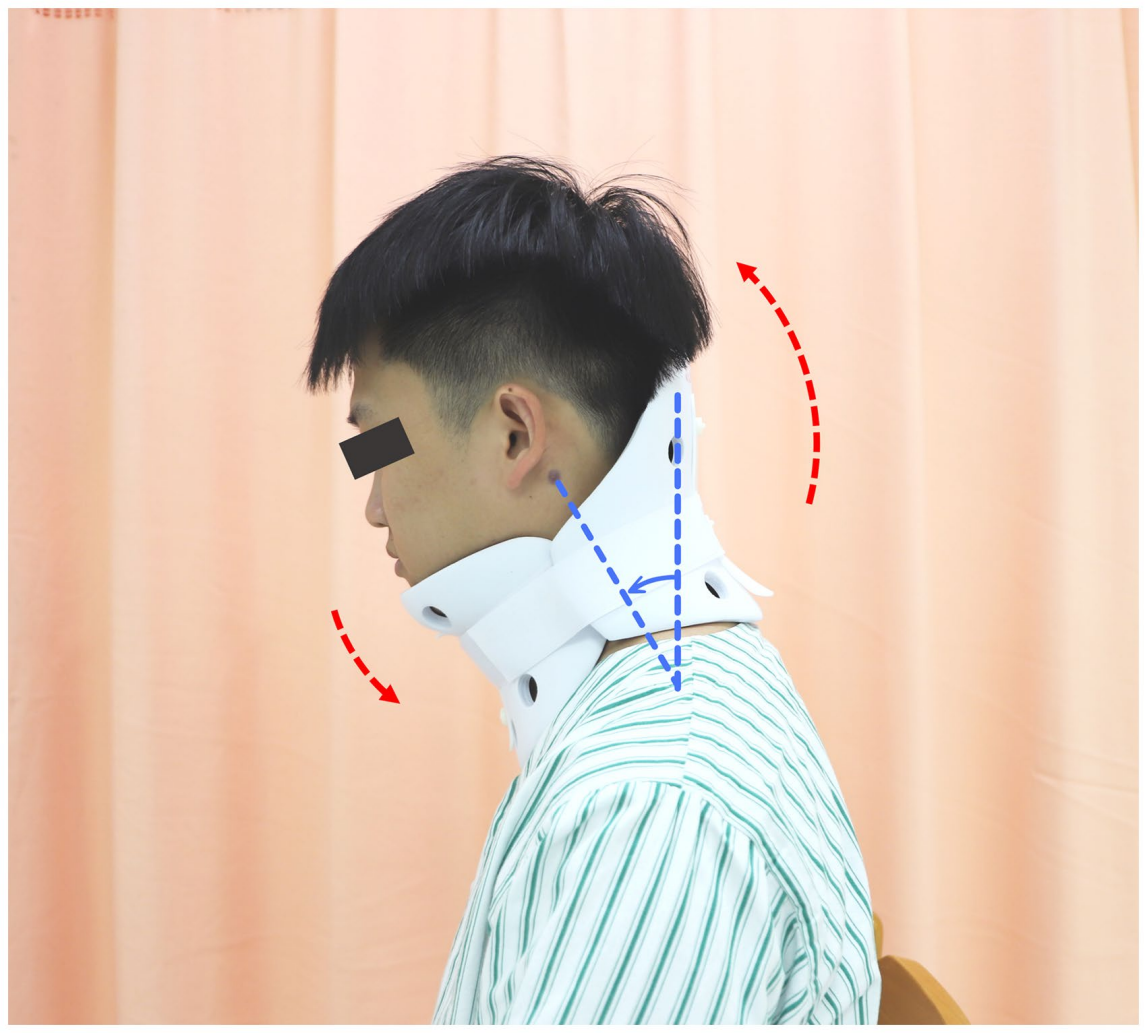
Illustration of wearing semi-hard cervical collars. Wearing a cervical collar immobilizes the participant’s neck in forward flexion.

Post-randomization, all participants will receive four-period usual care provided by the research physician, who will be independent of the outcome evaluation. The usual care in this trial will involve: (1) taking analgesics; (2) immobilizing the neck at a 10°- 20° anteflexion by wearing semi-hard cervical collars when ambulatory (Figure 3), and by inclining pillows while bedridden; and (3) as much bed rest as possible.

The analgesic diclofenac sodium (75 mg, extended-release) will be prescribed due to it being more readily available than other NSAIDs at GPHCM. Participants will be instructed to take 75 mg of diclofenac sodium orally once daily, with permission to adjust the dosage as needed based on their pain level, but not exceeding 150 mg per day. In cases where pain is poorly controlled despite the maximum daily dose of diclofenac sodium being administered, participants should be encouraged to take opiates with the guidance of the research physician. Each participant will be equipped with a specially-designed diary and required to note the following daily: (1) any analgesics they take; (2) collar-wearing hours; and (3) whether or not they were using inclining pillows. Any unused analgesics must be returned during the fifth-period visit. The quantity of returned analgesics will then be registered in the trial management documents.

Participants randomized to the control arm will receive usual care alone.

#### 3.5.2 AMTs

Participants randomized to the experimental group will receive 14 additional sessions of AMT over the four usual care periods, with one session per day in the first period, three sessions in the second period, and two sessions per period in the remaining two periods. It will be administered by a registered therapist who is blinded to the outcome evaluation. The therapist will be required to complete structured training with a self-developed AMT teaching robot (Patent No. CN202311394450.9, CN202311398626.8) and become familiar with the AMT procedure before participating in the pAMTLER.

AMT methodology

This composite intervention procedure begins with pressing-kneading manipulation, is followed by traction manipulation, and concludes with mechanical traction (Appendix S5).

**Figure 4:**
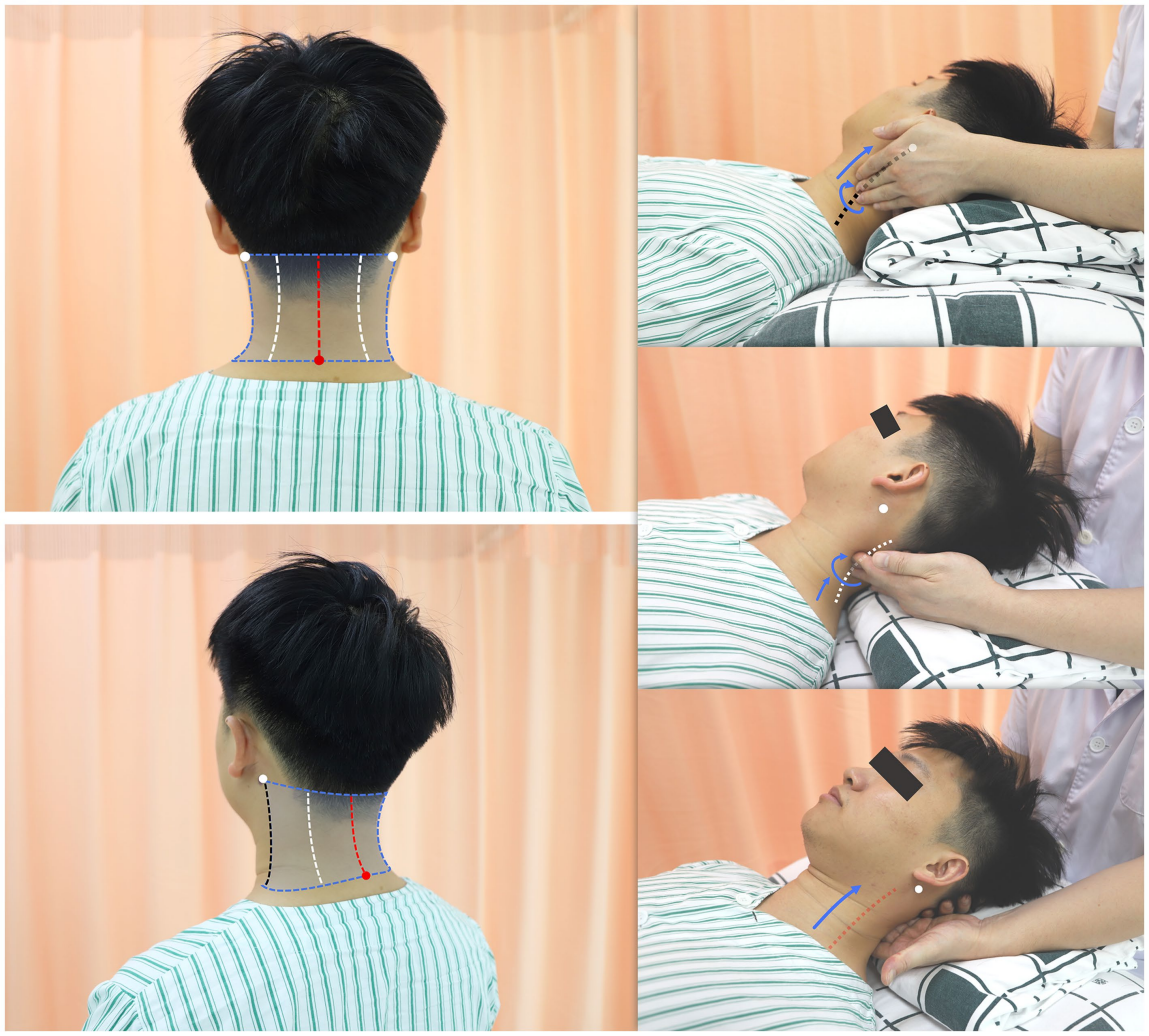
Illustration of pressing-kneading manipulation. *white dot*, mastoid process; *red dot*, the 7th cervical vertebra; *dashed blue box*, the nuchal region for pressing and kneading; *black dashed line*, transverse process line; *white dashed line*, zygapophyseal process line; red *dashed line*, pinous process line.

Pressing-kneading manipulation (Figure 4) refers to pressing and kneading the participant’s nuchal region, which is bounded by the 7th cervical vertebra horizontal level and the bilateral mastoid process vertical lines, with the neck anteriorly flexed. The participants will lie supine on the therapy table, with pillows used to gently elevate their heads and necks until the neck is anteriorly flexed to an angle that relieves pain or numbness in the cervicobrachial region. The therapist will sit on a chair at the cranial end of the therapy table, close to the participant’s head. With the palm of one hand supporting the participant’s occiput, the therapist will sequentially perform pressing-kneading along the transverse, zygapophyseal, and spinous process lines within the defined nuchal region using his other hand. He should use the pulps of the index, middle, and ring fingers to press and knead the nuchal muscles along these three process lines in an upward and inward direction, from lateral to medial. To alleviate any muscle tension around the nuchal region, the knead-pressing should be administered at a rate of 30 times per minute, with 3 repetitions on each side. The pressing-kneading force applied should be within the participants’ tolerance, allowing them to feel comfortable and relaxed. Additionally, any tender points identified during knead-pressing should be massaged at a frequency of 50 times every 30 s.

**Figure 5:**
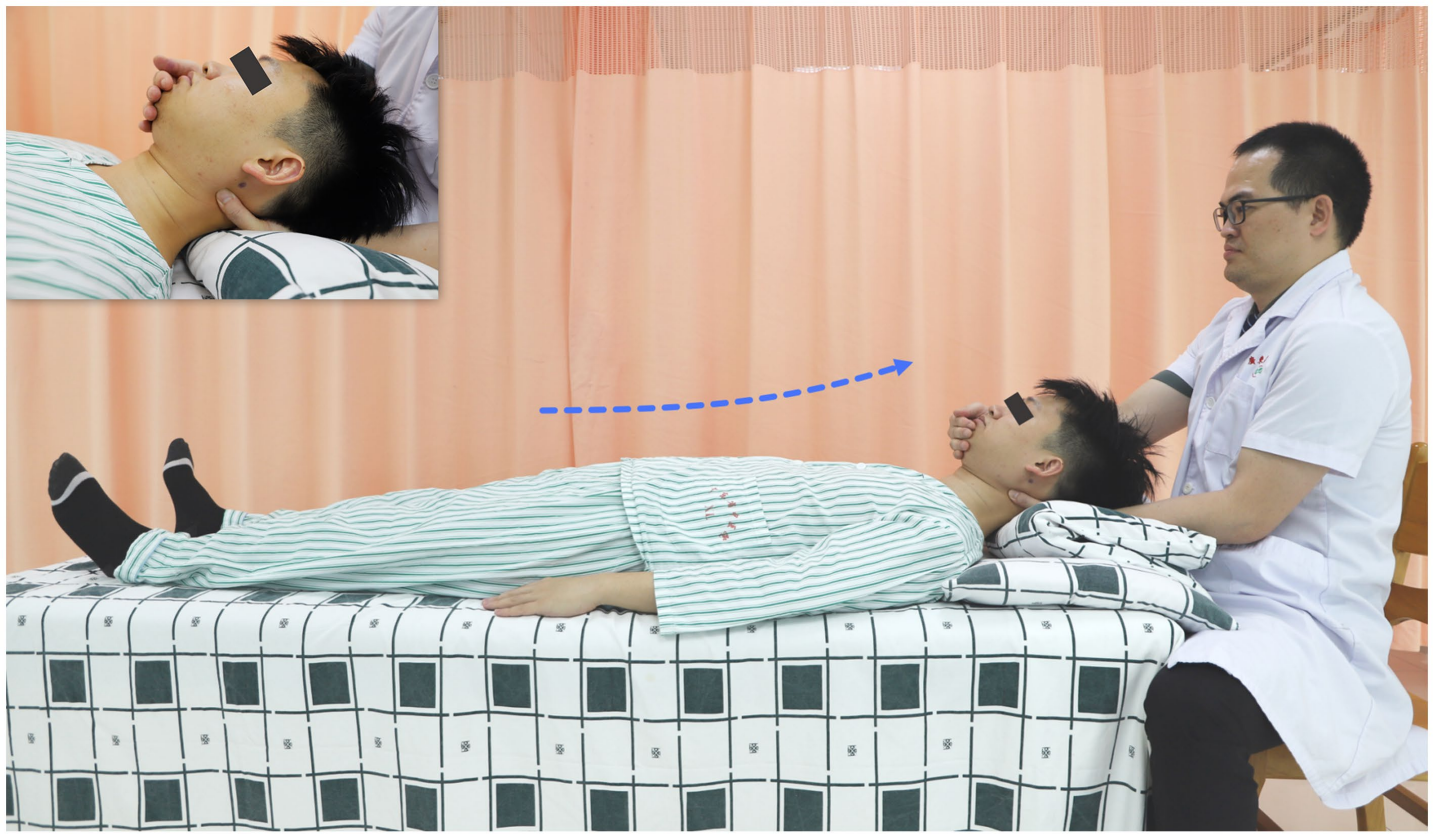
Illustration of traction manipulation. With the occiput and chin as contact points, apply a manual pulling force along the forward-flexion angle of the participant’s cervical spine, directed away from the body, until visible passive movement of the toes is observed.

Traction manipulation (Figure 5) involves using both hands to apply pulling force to the participant’s cervical spine along its longitudinal axis away from the body, with the neck anteriorly flexed to various angles. The therapist will first support the supine participant’s occiput with the palm of his dominant hand, positioning the thumb-index finger web space at the participant’s occipito-cervical junction. The therapist’s other hand will cradle the participant’s chin with the hypothenar. The held occiput and chin serve as the primary contact points to stabilize the participant’s head and neck at a controlled anterior flexion angle and to transmit the manual pulling force. By clamping the upper arms and adducting them toward the chest, the therapist transmits arm- and trunk-generated force through these contact points to produce traction, pulling the participant’s cervical spine away from the torso along the angle of anterior neck flexion. The traction angles will be determined by pathoanatomy causing nerve root compression, and will be dynamically adjusted to achieve patient-reported comfort or reduction in pain and/or numbness. For further details on the traction angles, see Table 1. The traction force will be incrementally increased until visible passive movement is observed in the patient’s toes. Each approximately 30-s traction will be followed by a 10- to 15-s rest interval, which will be repeated 5 times.

**Table 1:** Pulling angles during manual intermittent traction.

Pathoanatomy in neural foramen causing nerve root compression		Traction angles
ventral degenerative changes, e.g., hypertrophy of the uncovertebral joints	No vertebral segment consideration required	15° of anterior flexion
dorsal degenerative changes, e.g., hypertrophy of the zygapophyseal joints, herniation of the intervertebral disk	at levels above C4	15° of anterior flexion
	at C4/5	20° of anterior flexion
	at C5/6	25° of anterior flexion
	at C6/7	30° of anterior flexion

The angles in this table, summarized from long-term clinical practice, can be dynamically adjusted within the given ranges to achieve patient-reported comfort or relief of pain/numbness. When both ventral and dorsal changes coexist follow the angle recommended for dorsal changes.

**Figure 6:**
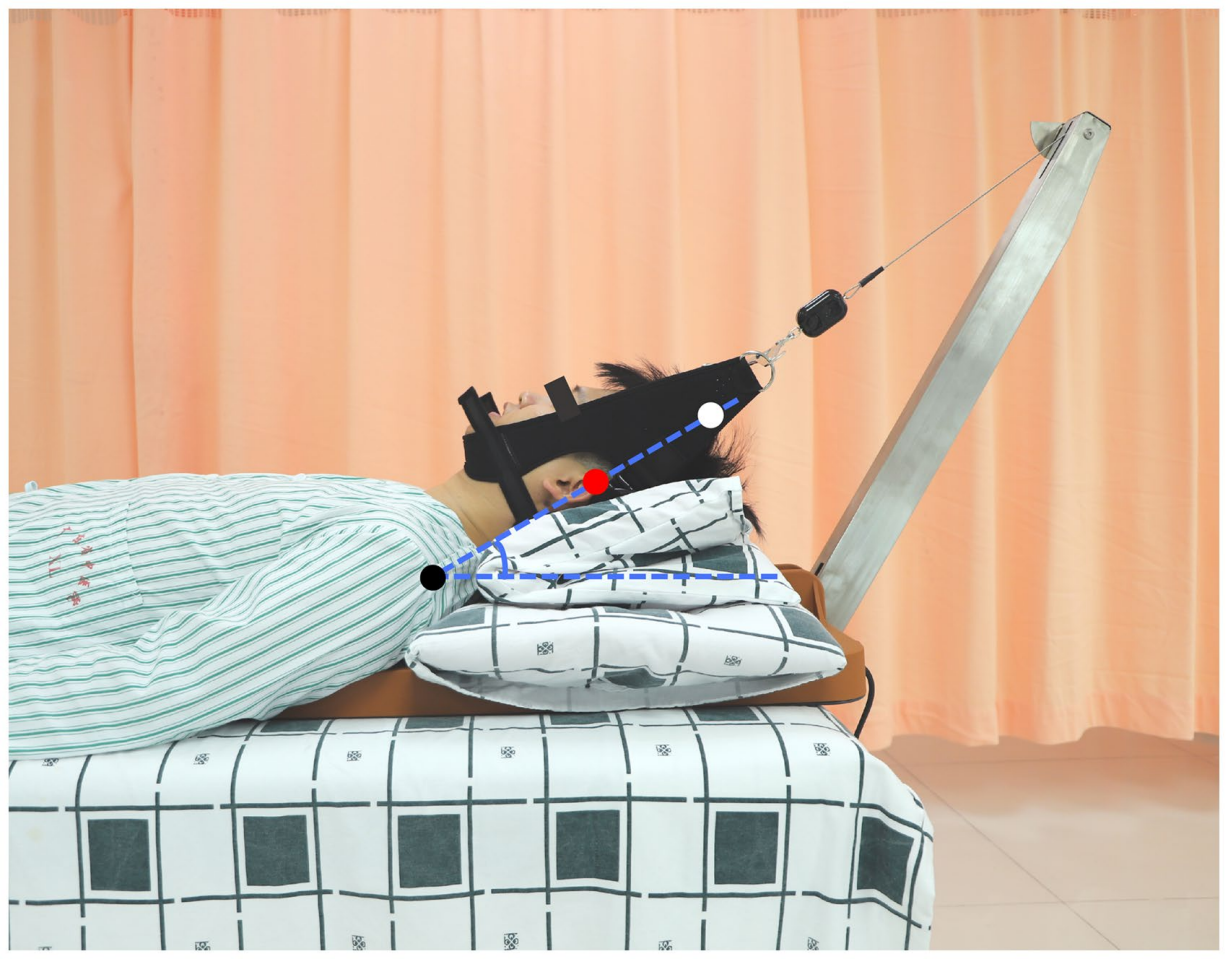
Illustration of mechanical traction. *Black dot*, acromion; *red dot*, ear apex; *white dot*, vertex. Adjust the pillows to straighten the line connecting the acromion, ear apex, and vertex such that the participant’s head–neck is in the supine position and has the same traction angle as during manual intermittent traction.

Following the first two *tuina* techniques, participants will be instructed to sit up gradually by assuming a lateral decubitus position. They will independently walk to and lie supine on another therapy table equipped with a self-developed motorized traction machine (Patent No. CN201720316913.3) to receive mechanical traction (Figure 6). Pillows will be used to elevate the supine participants’ heads and necks. They will then be adjusted until the line connecting the acromion, ear apex, and vertex becomes straight, such that the head–neck traction angle is consistent with that during traction manipulation. Meanwhile, a soft cushion will be placed at the cervicothoracic junction to prevent it from becoming unsupported. When the position and angle for mechanical traction is ready, the participants’ heads will be secured to a traction belt in the motorized traction device, and a traction force determined based on their body weight will be gently applied. The traction force will be approximately 10% of the participants’ body weight, typically ranging from 6 kg to 8 kg. This pulling force will be maintained for about 15 min, but will be adjustable according to the participants’ comfort.

#### 3.5.3 Prohibited therapies

Any therapies that might alleviate cervicobrachial pain beyond usual care will be prohibited during study participation, including dehydrating agents, neurotrophic drugs, skeletal muscle relaxants, Chinese herbal medicine, acupuncture, cupping, physical therapy and exercise therapy. Participants who consider pain reduction to be insufficient may seek alternative therapeutic options, but will be withdrawn from the trial.

### 3.6 Outcomes

#### 3.6.1 Primary outcome measurements

Measures that indicate the feasibility of the future full-scale RCT will serve as primary outcomes. They will be:

1. enrolment rate: the proportion of recruited participants who are randomized.
2. weekly enrolment rate: the number of participants to be randomized per week over the trial recruitment duration.
3. drop-out rates: the proportion of participants who either withdraw or are lost to follow-up at the initial visits of the 2nd, 3rd, 4th, or 5th periods.
4. retention rates: the proportion of non-dropout participants (i.e., those completing all follow-up measurements) at the initial visits of the 2nd, 3rd, 4th, and 5th periods.
5. adherence rates: the proportion of participants completing the planned number of treatment sessions after randomization (the proportion completing 14 sessions of AMT in the experimental group and attending 4 usual care prescriptions in the control group, respectively).
6. completion rates of efficacy-related outcome measures: the proportion of completed efficacy outcome measures relative to those intended for assessment at the initial visit per period.
7. completion rates of cost-related outcome measures: the proportion of completed cost outcome measures relative to those intended for assessment at every visit during the trial.

#### 3.6.2 Secondary outcome measurements

Measurements intended for efficacy assessment in the future definitive trial will be designated as the secondary outcomes. These secondary outcome measures are scheduled to be assessed prior to treatment administration during each period’s initial visit (as shown in Figure 2), and outlined below:

1. pain rating with the NRS, including cervicobrachial pain, upper extremity pain, neck pain, and shoulder pain.
2. numbness in the upper extremities graded by the NRS.
3. muscle weakness recorded by grip strength using a hand dynamometer (CAMRY EH101) in kilograms.
4. upper extremity disability assessed by the quick disabilities of the arm, shoulder, and hand questionnaire (QuickDASH)
5. neck disability scoring with the neck disability index (NDI)
6. NSAID and opiate consumption per period documented in patient diaries.
7. work-productivity impairment evaluated with the work productivity and activity impairment questionnaire: cervical radiculopathy (WPAI: CR)
8. quality of life quantified with the 5-level version of the euroqol 5-dimensional descriptive system (EQ-5D-5L)
9. emotional well-being measured by Zung’s self-rating depression scale (SDS) and Zung’s self-rating anxiety scale (SAS)

The NRS is a horizontal bar or line evenly segmented by 11 integers, in which “0” on the far left represents “no pain” while “10” on the far right indicates “the worst pain imaginable” (34). It is a popular scale for quantifying pain intensity without usage barriers across cultures and languages (35), and has also been well-validated among patient populations with musculoskeletal pain (36–40). Participants will be instructed to circle one number on the NRS to represent the amount of pain that they are experiencing at the time of evaluation (40). Clinically meaningful pain relief can be concluded when a reduction of at least 30% or 2 points is observed on the NRS (41). Given its recommendation for assessing non-pain subjective experiences, we employed NRS in our study to quantify numbness in the upper extremities (42).

The NDI is an extensively tested self-rated scale for diminished neck function due to pain-related conditions in everyday activities (43). This patient-based questionnaire consists of 10 items scored from 0 to 5, with higher scores corresponding to greater perceived handicaps (44). The total score can be reported as raw points from 0 to 50, or as a percentage of the maximal raw points (45, 46). A validated Chinese version of the NDI will be used in this trial (46), with raw points serving as the scoring method (45). Items on the NDI that are not applicable to respondents’ lives may be omitted, but no more than two. These missing items will be replaced by the average score of the answered items (45). 7 points has been suggested as the minimum clinically important difference (MCID) for NDI among populations with CR (47, 48).

The QuickDASH is a well-recognized patient-reported outcome measure designed to determine the extent to which upper-extremity incapacities impact daily routines, social events, and recreational activities (49, 50). It contains 11 items (3 for symptoms and 8 for function) scored from 1 to 5 on a 5-point Likert scale, with an optional additional module that will be omitted in this trial due to its rare use in patient settings (51). Among the 11 items, only one item related to function can be missing (52). The score of the answered items in QuickDASH can then be converted into a 0-to-100 scaled score using an e-tool provided on the official website (53). A greater converted value indicates greater perceived upper-extremity impairment. The Chinese simplified version QuickDASH (54, 55) with a recommended 14-point MCID (56) will be used in our trial.

The EQ-5D-5L is a readily available generic health status instrument which has shown reliability and validity on musculoskeletal problems (57). It consists of a visual analog scale (EQ-VAS) from 0 to 100 and a five-item descriptive system, with each item having five response levels of severity (58). The digits which participants mark on the EQ-VAS represent their perceived overall health on the day of being interviewed. The five ticked response levels in the descriptive system can be derived into a 0-to-1 index value employing a country-specific ‘value set’ to express a health state profile. Higher EQ-VAS and EQ-5D-5L index values both reveal a better quality of life. In our trial, a Chinese version of EQ-5D-5L obtained from the official website (59) and the China-specific ‘value set’ (60) will be applied. However, the EQ-5D-5L index values will be converted into quality-adjusted life years (QALYs) to inform economic evaluations of interventions.

The SDS (61) and SAS (62) are self-administered instruments for rating depression and anxiety severity based on the frequency of affective and somatic symptoms during the preceding week. Each contains 20 questions scored from 1 to 4 on a 4-point Likert scale based on the perceived frequency, all yielding a raw summation range of 20 to 80. The raw summations will be converted into index scores by multiplying them by 1.25. The index scores can categorize depression as nil (25–49), mild (50–59), moderate (60–69), or severe (70+), and anxiety as nil (25–44), mild (45–59), moderate (60–74), or severe (75+) (63). The Chinese versions (64, 65) which have been demonstrated to have extensive reliability and validity among the Chinese population (66, 67) will be administered in our trial.

The WPAI: CR is a revised questionnaire constructed by specifying “cervical radiculopathy (CR)” in the Work Productivity and Activity Impairment Questionnaire: Specific Health Problem V2.0 (WPAI: SHP). The WPAI: SHP is a six-item patient-reported instrument created for capturing the amount of work time missed, on-the-job effectiveness reduced, and daily activity impairment during the previous 7 days due to a specific health problem (68, 69). The official user guide encourages adapting the WPAI: SHP to any specific health problem by inserting the name of the disease or condition of interest wherever the word ‘PROBLEM’ appears in the WPAI: SHP template (70). In our trial, CR-related work productivity loss will be quantified based on the revised WPAI: CR through per cent work time missed, impairment while working, overall work impairment and activity impairment (71). Greater values indicate less productivity.

Additional secondary outcome measures will include adverse event rate (AER), adverse reaction rate (ARR) and intervention cost. Any undesirable experiences from which participants suffer post-enrolment will be identified as adverse events (AE), and those associated with the intervention will be defined as adverse reactions, while those that result in hospitalization, loss of ability to work, disability, congenital deformity, or death will be listed as serious adverse events (SAE). Any AE during the study will be recorded. The cost of intervention will be based on a societal perspective, and composed of direct medical costs (DMC), direct non-medical costs (DnMC) and indirect costs (IDC) (72). The composition of direct costs are detailed in Table 2, and the composed item cost will be obtained by resource volumes multiplied by their respective unit costs (73). The IDC will be estimated using the human capital approach, which is the product of labor loss time and the unit labor value (72–74). In this trial, labor loss time will encompass the work time missed by both participants and their accompanying family members. The unit labor value will be based on the per capita GDP of Guangzhou in 2024 (73). DMC, DnMC, and the accompanying family’s missed work time will be collected at every visit. The participants’ missed work time will be collected via the WPAI: CR.

**Table 2:** Composition and determination of direct costs.

Cost composition	Basis for unit cost calculation
DMC resulting from registration, magnetic resonance imaging, NSAIDs and opiates, cervical collars, and AMTs	price summary list of basic medical services in Guangzhou
DMC resulting from adverse reactions	actual costs incurred during the trial
DnMC resulting from round-trip travel, meal and lodging	actual costs incurred during the trial

### 3.7 Statistical methods

#### 3.7.1 Sample size

The primary objective of the pAMTLER is to determine the feasibility of a future definitive full-scale RCT based on recruitment rate, retention rate, and intervention acceptability. With pre-specified feasibility thresholds of ≥25% recruitment, ≥80% retention and ≥80% adherence, at least 48 eligible participants are required to have a 95% confidence interval with a maximum width of 20% (75). This sample size, in a resource-limited setting, is considered adequate to provide a reasonable estimate of outcome variability across the four strata (12 participants per stratum), defined by sex and baseline cervicobrachial pain level (75).

#### 3.7.2 Data analysis

Data will be statistically analyzed using PASW Statistics 18.0, STATA 14.0 and R 4.3.0. Continuous variables will be summarized as means with standard deviations or medians with interquartile ranges, whereas categorical variables will be presented as counts and percentages. The primary outcome will be outlined using descriptive statistics. Building on reported positive associations between colder temperatures, male sex, and more consultations for musculoskeletal pain (76), we will conduct subgroup analyses to explore how season and sex influence enrolment, retention, and adherence, thereby informing weather-adaptive management strategies for the future full RCT. Secondary outcome measures will be analyzed based on the intention-to-treat population approach. Any post-randomization participants who received at least one intervention session will be included in the full analysis set. Among these, those who completed at least 80% of the planned treatments without any significant protocol violations will be categorized into the per-protocol analysis set. Any post-randomization participants receiving at least one intervention session will be classified into the safety analysis set. Any missing data will primarily be handled using multiple imputation, with last observation carried forward applied for sensitivity analysis.

We will analyze both within- and between-group changes for each efficacy-related secondary outcome variable using a linear mixed-effects model to derive estimates of intervention effects.

Group allocation, time-point and the interaction between group allocation and time-point will act as fixed-effects, individuals as random effects, and baseline measurements as covariates. Given that this exploratory trial is not formally powered to assess effects, a recommended confidence interval of 75% will be employed to infer the size and direction of the calculated effect estimates (77). The 75% confidence interval will be compared with the MCIDs to preliminarily assess whether the difference between treatment groups holds potential for clinical significance (77). The remaining secondary outcome measures involving AER, ARR, and intervention costs, as well as baseline variables, will be presented descriptively by the groups.

### 3.8 Trial management and quality control

A trial management group (TMG), a trial steering committee (TSC) and standard operating procedures related to participates, informed consent enrolment, intervention, outcome evaluation and data collection will be established prior to trial commencement. Given the pilot and feasibility nature of our trial with short-term follow-up, an independent data monitoring committee will not be established.

The TMG assumes responsibility for day-to-day trial operations. It will consist of a project manager, a registered research physician, a registered therapist, a trial coordinator, two research assistants and a data manager. All members will attend a pre-trial training workshop to familiarize themselves with the trial implementation process, and to ensure adherence to the study protocol and standard operating procedures. The trial coordinator is to formulate follow-up programs based on the study protocol. They will keep in touch with participants to facilitate their attendance at scheduled follow-ups, but also to assist in promptly detecting any AE. The research assistants will collect and record data on paper case report forms (CRF), including participants’ socio-demographics (age, sex, education level, employment status, health insurance, etc.), medical history (comorbidities, concomitant medications, details related to the cervicobrachial pain episode, etc.) and physical examination at baseline (dominant hand, abnormal deep tendon reflexes, radiculopathy level shown on magnetic resonance imaging, etc.) and discharge (abnormal deep tendon reflexes), secondary outcomes, treatment information, and adverse event details. Data may be collected within 1 day after the scheduled time. Unless they explicitly refuse, research assistants will follow up with any withdrawn participants to acquire data until the trial end. Additionally, they will track any AE from reporting until resolution. The data manager will regularly monitor data quality, working with research assistants to recover missing data and clarify ambiguous data to ensure all reported data are accurate, complete, legible and verifiable from source documents. Research assistants will double-enter the data from the paper CRF after monitoring into an Epidata-based electronic data capture system. Any discrepancies from double entry will be verified against the paper CRF. The data manager will lock the electronic data capture after all data have been cleaned; researchers will be unable to modify the data. The TMG will meet weekly to review study progress during the trial. The project manager will meet with the TSC biweekly to align on key decisions.

The TSC will consist of a principal investigator (Dr Dingkun Lin), a clinical research methodologist (Prof. Zehuai Wen), a spinal surgery expert experienced in clinical trials (Dr Yongjin Li) and the following independent members: a consultant orthopaedist and chair (Dr Guoyi Su), a CR patient, and a public involvement representative. Besides approving the study protocol and the trial’s operational guidelines, the TSC will regularly convene to monitor protocol compliance in the administered trial, review the safety data, and resolve any problems from the TMG. Any SAE will be reported to the TSC post-haste, and submitted to the GPHCM Ethics Committee within 24 h. The TSC has the discretion to discontinue any patient’s participation in the study due to safety precautions.

## 4 Results

Recruitment for the pAMTLER began on 1 August 2024. Thirty-three patients have been recruited as of 31 July, 2025. The trial was originally scheduled to end in August 2025. However, due to slow recruitment, the trial may have to be extended to late June 2026. We expect participant recruitment to conclude in late December 2025, data collection to be finished in late February 2026, and results to be available and published in late June 2026.

Successful pilot targets are defined as a ≥ 25% enrolment rate, a ≥ 80% adherence rate as well as a ≥ 80% retention rate (78, 79). The trial may be infeasible if enrolment is <15%, adherence is <70%, or retention is <70%. Therefore, strong measures should be taken to improve feasibility. Additionally, we hypothesize that the AMT add-on arm will result in better health outcomes than the usual care control arm. The study results will be disseminated through open-access peer-reviewed journals, scientific conferences, and public channels.

## 5 Discussion

A full RCT with appropriate power is necessary to establish the clear effects of AMT on pain relief and functional improvement in patients with CR. However, such studies conducted over the past decade have been insufficient. Therefore, establishing whether a more extensive future RCT would be viable is of paramount importance (78). The pAMTLER study is a randomized pilot developed to address this issue. It will focus on assessing the ability to enroll CR individuals with moderate-to-severe cervicobrachialgia, to retain participants for the intervention duration, and to encourage participants to adhere to the protocol during the trial, thereby establishing the feasibility of a proposed full-scale RCT. Meanwhile, the pAMTLER study can provide preliminary data on the efficacy and safety of AMT for CR, thereby complementing the key estimates regarding outcome variability necessary for sample size calculations in the confirmatory trial.

Several advantages make the pAMTLER study instructive for a future definitive RCT on AMT for CR. To begin, the pAMTLER study meets the methodological requirements for feasibility and pilot projects. It was carefully designed, not only with adequate randomization, allocation concealment, and blinding of outcome assessors and statisticians, but also specifying clear feasibility objectives, analytic plans, and criteria for the success of feasibility (80). Additionally, with small feasibility study samples, confidence intervals for clinically important differences in outcomes of interest are narrowed to a 75% confidence level. Such analytical strategies are justified, contributing to realistic and unbiased effect estimates to inform subsequent power calculation for the future full definitive RCT (77, 79). Third, unlike most previous RCTs which directly compare different multimodal conservative strategies for CR patients (20, 81), the pAMTLER study is characterized by a randomized add-on trial. An add-on study design could be more rational in circumstances where analgesics are commonly preferred as the first-line therapy (82), while also allowing for further understanding of AMT’s superiority in real-world clinical practice of CR.

However, there are still limitations that should be disclosed. The first is failing to blind the participants, the therapist and the research physician. Since most outcomes are measured using patient self-reported instruments, the fact that these individuals remain unblinded may introduce measurement bias. To address this, participants in each arm will be escorted by a trial coordinator to a dedicated clinic room for visits and interventions. The therapist will be barred from contact with control-arm participants and from outcome-related data collection or evaluation; the research physician, remaining neutral, will inquire only about pain improvement and will likewise refrain from any outcome-related assessment. To reduce selection bias arising from the unmasked research physician, a structured eligibility screening process will be implemented. Also, the research physician will be isolated from intervention allocation. The inability to conduct a process evaluation to further assess the fidelity, adherence, contextual factors, implementation facilitators of and barriers to AMT and usual care following the initiative might represent another limitation. Given the constrained research funding for the pAMTLER, a process evaluation will be conducted in parallel with the future well-funded full-scale RCT.

## 6 Conclusion

The pAMTLER is an essential precursor for the development of a future full-scale definitive RCT. If successful, the formative data provided by this external pilot study will be used to design a fully powered RCT to demonstrate the clinical efficacy and cost-effectiveness of AMT within a population with CR with moderate-to-severe cervicobrachialgia.

## Glossary

AMT: angled manual traction
CR: cervical radiculopathy
RCT: randomized controlled trial
NSAIDs: non-steroidal anti-inflammatory drugs
GPHCM: Guangdong Provincial Hospital of Chinese Medicine
QuickDASH: quick disabilities of the arm, shoulder, and hand questionnaire
NDI: neck disability index
WPAI: CR: work productivity and activity impairment questionnaire (cervical radiculopathy)
WPAI: SHP: Work Productivity and Activity Impairment Questionnaire: Specific Health Problem V2.0
EQ-5D-5L: 5-level version of the euroqol 5-dimensional descriptive system
SDS: Zung’s self-rating depression scale
SAS: Zung’s self-rating anxiety scale
MCID: minimum clinically important difference
AER: adverse event rates
ARR: adverse reaction rate
AE: adverse events
SAE: serious adverse events
DMC: direct medical costs
DnMC: direct non-medical costs
IDC: indirect costs
TMG: trial management group
TSC: trial steering committee
