# Supplementary material for: Angled manual traction and usual care for cervical radiculopathy: rationale and a protocol for a pilot randomized controlled trial (pAMTLER)
Source: Front Med (Lausanne). 2025 Dec 19;12:1695623. doi: 10.3389/fmed.2025.1695623 (PMC12757876; doi:10.3389/fmed.2025.1695623)
Supplement: Supplementary file 1 [file Data_Sheet_1.pdf]

Supplementary Material

1 S1 Appendix. Preliminary biomechanical findings of AMT (Ongoing and Not Yet Fully Disclosed)

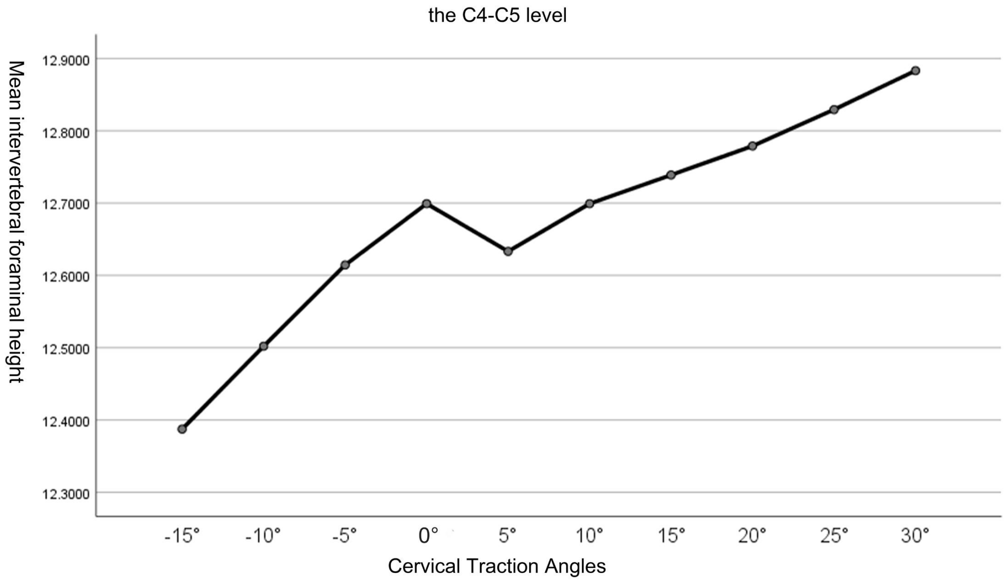

Mean intervertebral foraminal height at the C4－C5 level under a 60 N axial tensile load in fresh-frozen cervical cadaveric specimens, measured at different cervical traction angles, including posterior extension (-15° to -5°), neutral position (0°), and anterior flexion (5° to 30°). The foraminal height increased progressively with greater anterior flexion angles.

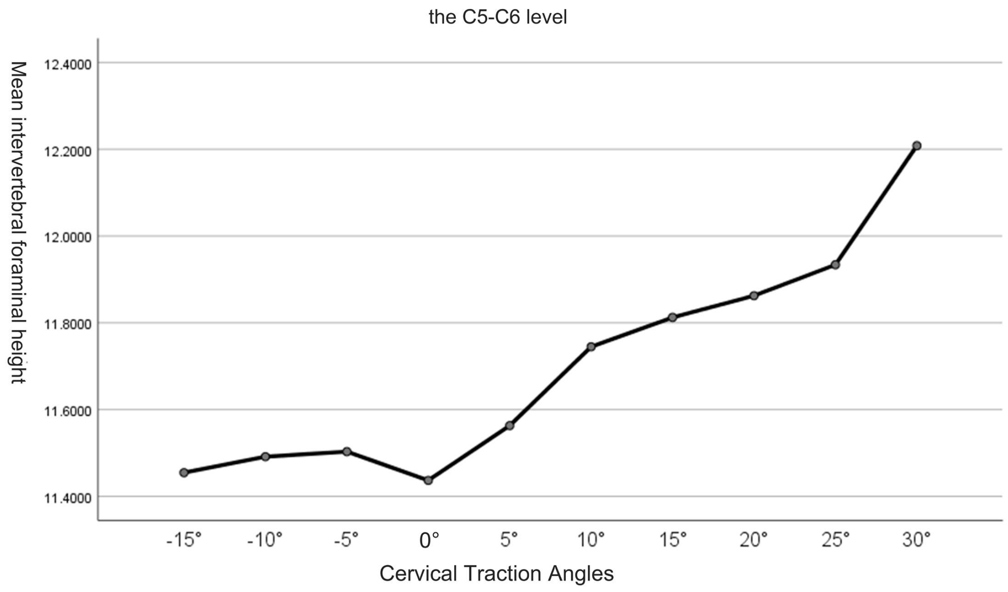

Mean intervertebral foraminal height at the C5－C6 level under a 60 N axial tensile load in fresh-frozen cervical cadaveric specimens, measured at different cervical traction angles, including

posterior extension (-15° to -5°), neutral position (0°), and anterior flexion (5° to 30°). The foraminal height increased progressively with greater anterior flexion angles.

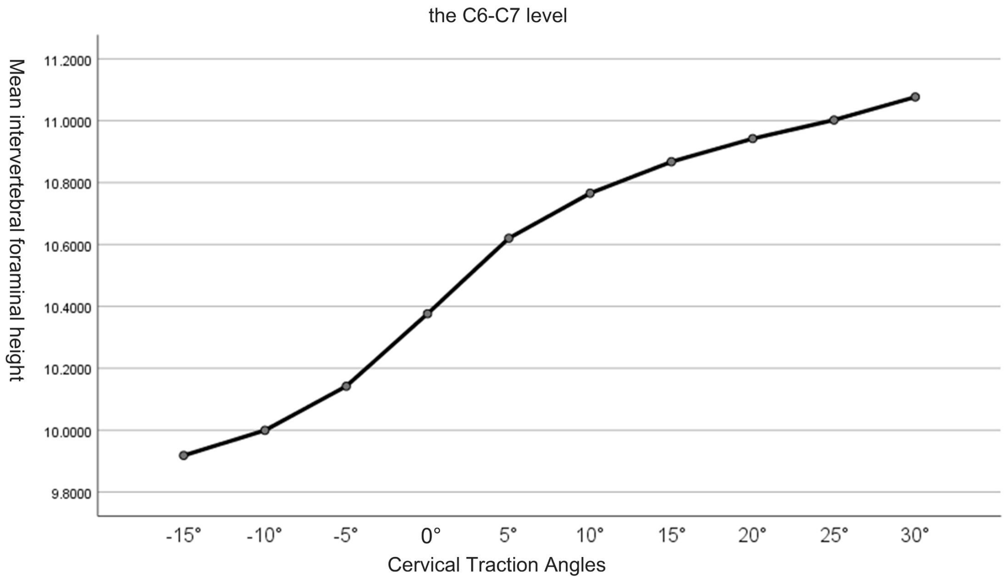

Mean intervertebral foraminal height at the C5–C6 level under a 60 N axial tensile load in fresh-frozen cervical cadaveric specimens, measured at different cervical traction angles, including posterior extension (-15° to -5°), neutral position(0°), and anterior flexion (5° to 30°). The foraminal height increased progressively with greater anterior flexion angles.

2 S2 Appendix. SPIRIT checklist

STANDARD PROTOCOL ITEMS: RECOMMENDATIONS FOR INTERVENTIONAL TRIALS

SPIRIT 2013 Checklist: Recommended items to address in a clinical trial protocol and related documents*

Section/item	Item No	Description	Page Number on which item is reported
**Administrative information**			
Title	1	Descriptive title identifying the study design, population, interventions, and, if applicable, trial acronym	1
Trial registration	2a	Trial identifier and registry name. If not yet registered, name of intended registry	2
	2b	All items from the World Health Organization Trial Registration Data SetWHO WHO	Not applicable
Protocol version	3	Date and version identifier	Not applicable
Funding	4	Sources and types of financial, material, and other support	15
Roles and responsibilities	5a	Names, affiliations, and roles of protocol contributors	1, 14-15
	5b	Name and contact information for the trial sponsor	15
	5c	Role of study sponsor and funders, if any, in study design; collection, management, analysis, and interpretation of data; writing of the report; and the decision to submit the report for publication, including whether they will have ultimate authority over any of these activities	15
	5d	Composition, roles, and responsibilities of the coordinating centre, steering committee, endpoint adjudication committee, data management team, and other individuals or groups overseeing the trial, if applicable (see Item 21a for data monitoring committee)	12-13
**Introduction**			

Background and rationale	6a	Description of research question and justification for undertaking the trial, including summary of relevant studies (published and unpublished) examining benefits and harms for each intervention	3-4
	6b	Explanation for choice of comparators	3
Objectives	7	Specific objectives or hypotheses	4
Trial design	8	Description of trial design including type of trial (eg, parallel group, crossover, factorial, single group), allocation ratio, and framework (eg, superiority, equivalence, noninferiority, exploratory)	5
**Methods: Participants, interventions, and outcomes**			
Study setting	9	Description of study settings (eg, community clinic, academic hospital) and list of countries where data will be collected. Reference to where list of study sites can be obtained	5
Eligibility criteria	10	Inclusion and exclusion criteria for participants. If applicable, eligibility criteria for study centres and individuals who will perform the interventions (eg, surgeons, psychotherapists)	5-6
Interventions	11a	Interventions for each group with sufficient detail to allow replication, including how and when they will be administered	7-8
	11b	Criteria for discontinuing or modifying allocated interventions for a given trial participant (eg, drug dose change in response to harms, participant request, or improving/worsening disease)	8
	11c	Strategies to improve adherence to intervention protocols, and any procedures for monitoring adherence (eg, drug tablet return, laboratory tests)	7, 12
	11d	Relevant concomitant care and interventions that are permitted or prohibited during the trial	8
Outcomes	12	Primary, secondary, and other outcomes, including the specific measurement variable (eg, systolic blood pressure), analysis metric (eg, change from baseline, final value, time to event), method of aggregation (eg, median, proportion), and time point for each outcome. Explanation of the clinical relevance of chosen efficacy and harm outcomes is strongly recommended	8-11

Participant timeline	13	Time schedule of enrolment, interventions (including any run-ins and washouts), assessments, and visits for participants. A schematic diagram is highly recommended (see Figure)	Fig 2
Sample size	14	Estimated number of participants needed to achieve study objectives and how it was determined, including clinical and statistical assumptions supporting any sample size calculations	11
Recruitment	15	Strategies for achieving adequate participant enrolment to reach target sample size	6
**Methods: Assignment of interventions (for controlled trials)**			
Allocation:			
Sequence generation	16a	Method of generating the allocation sequence (eg, computer-generated random numbers), and list of any factors for stratification. To reduce predictability of a random sequence, details of any planned restriction (eg, blocking) should be provided in a separate document that is unavailable to those who enrol participants or assign interventions	6
Allocation concealment mechanism	16b	Mechanism of implementing the allocation sequence (eg, central telephone; sequentially numbered, opaque, sealed envelopes), describing any steps to conceal the sequence until interventions are assigned	6
Implementati on	16c	Who will generate the allocation sequence, who will enrol participants, and who will assign participants to interventions	6
Blinding (masking)	17a	Who will be blinded after assignment to interventions (eg, trial participants, care providers, outcome assessors, data analysts), and how	6
	17b	If blinded, circumstances under which unblinding is permissible, and procedure for revealing a participant's allocated intervention during the trial	Not Applicable
**Methods: Data collection, management, and analysis**			
Data collection methods	18a	Plans for assessment and collection of outcome, baseline, and other trial data, including any related processes to promote data quality (eg, duplicate measurements, training of assessors) and a description of study instruments (eg, questionnaires, laboratory tests) along with their reliability and validity, if known. Reference to where data collection forms can be found, if not in the protocol	9-11, 12

	18b	Plans to promote participant retention and complete follow-up, including list of any outcome data to be collected for participants who discontinue or deviate from intervention protocols	12
Data management	19	Plans for data entry, coding, security, and storage, including any related processes to promote data quality (eg, double data entry; range checks for data values). Reference to where details of data management procedures can be found, if not in the protocol	12
Statistical methods	20a	Statistical methods for analysing primary and secondary outcomes. Reference to where other details of the statistical analysis plan can be found, if not in the protocol	11-12
	20b	Methods for any additional analyses (eg, subgroup and adjusted analyses)	Not applicable
	20c	Definition of analysis population relating to protocol non-adherence (eg, as randomised analysis), and any statistical methods to handle missing data (eg, multiple imputation)	11-12
**Methods: Monitoring**			
Data monitoring	21a	Composition of data monitoring committee (DMC); summary of its role and reporting structure; statement of whether it is independent from the sponsor and competing interests; and reference to where further details about its charter can be found, if not in the protocol. Alternatively, an explanation of why a DMC is not needed	12
	21b	Description of any interim analyses and stopping guidelines, including who will have access to these interim results and make the final decision to terminate the trial	Not applicable
Harms	22	Plans for collecting, assessing, reporting, and managing solicited and spontaneously reported adverse events and other unintended effects of trial interventions or trial conduct	11, 12, 13
Auditing	23	Frequency and procedures for auditing trial conduct, if any, and whether the process will be independent from investigators and the sponsor	12-13
**Ethics and dissemination**			

Research ethics approval	24	Plans for seeking research ethics committee/institutional review board (REC/IRB) approval	5, S4
Protocol amendments	25	Plans for communicating important protocol modifications (eg, changes to eligibility criteria, outcomes, analyses) to relevant parties (eg, investigators, REC/IRBs, trial participants, trial registries, journals, regulators)	Not applicable
Consent or assent	26a	Who will obtain informed consent or assent from potential trial participants or authorised surrogates, and how (see Item 32)	6
	26b	Additional consent provisions for collection and use of participant data and biological specimens in ancillary studies, if applicable	Not applicable
Confidentiality	27	How personal information about potential and enrolled participants will be collected, shared, and maintained in order to protect confidentiality before, during, and after the trial	12
Declaration of interests	28	Financial and other competing interests for principal investigators for the overall trial and each study site	14
Access to data	29	Statement of who will have access to the final trial dataset, and disclosure of contractual agreements that limit such access for investigators	14
Ancillary and post-trial care	30	Provisions, if any, for ancillary and post-trial care, and for compensation to those who suffer harm from trial participation	Not applicable
Dissemination policy	31a	Plans for investigators and sponsor to communicate trial results to participants, healthcare professionals, the public, and other relevant groups (eg, via publication, reporting in results databases, or other data sharing arrangements), including any publication restrictions	13
	31b	Authorship eligibility guidelines and any intended use of professional writers	Not applicable
	31c	Plans, if any, for granting public access to the full protocol, participant-level dataset, and statistical code	Not applicable
**Appendices**			
Informed consent materials	32	Model consent form and other related documentation given to participants and authorised surrogates	Not applicable

Biological specimens	33	Plans for collection, laboratory evaluation, and storage of biological specimens for genetic or molecular analysis in the current trial and for future use in ancillary studies, if applicable	Not applicable

*It is strongly recommended that this checklist be read in conjunction with the SPIRIT 2013 Explanation & Elaboration for important clarification on the items. Amendments to the protocol should be tracked and dated. The SPIRIT checklist is copyrighted by the SPIRIT Group under the Creative Commons "Attribution-NonCommercial-NoDerivs 3.0 Unported" license.

3 S3 Appendix. CONSORT diagram of participant flow

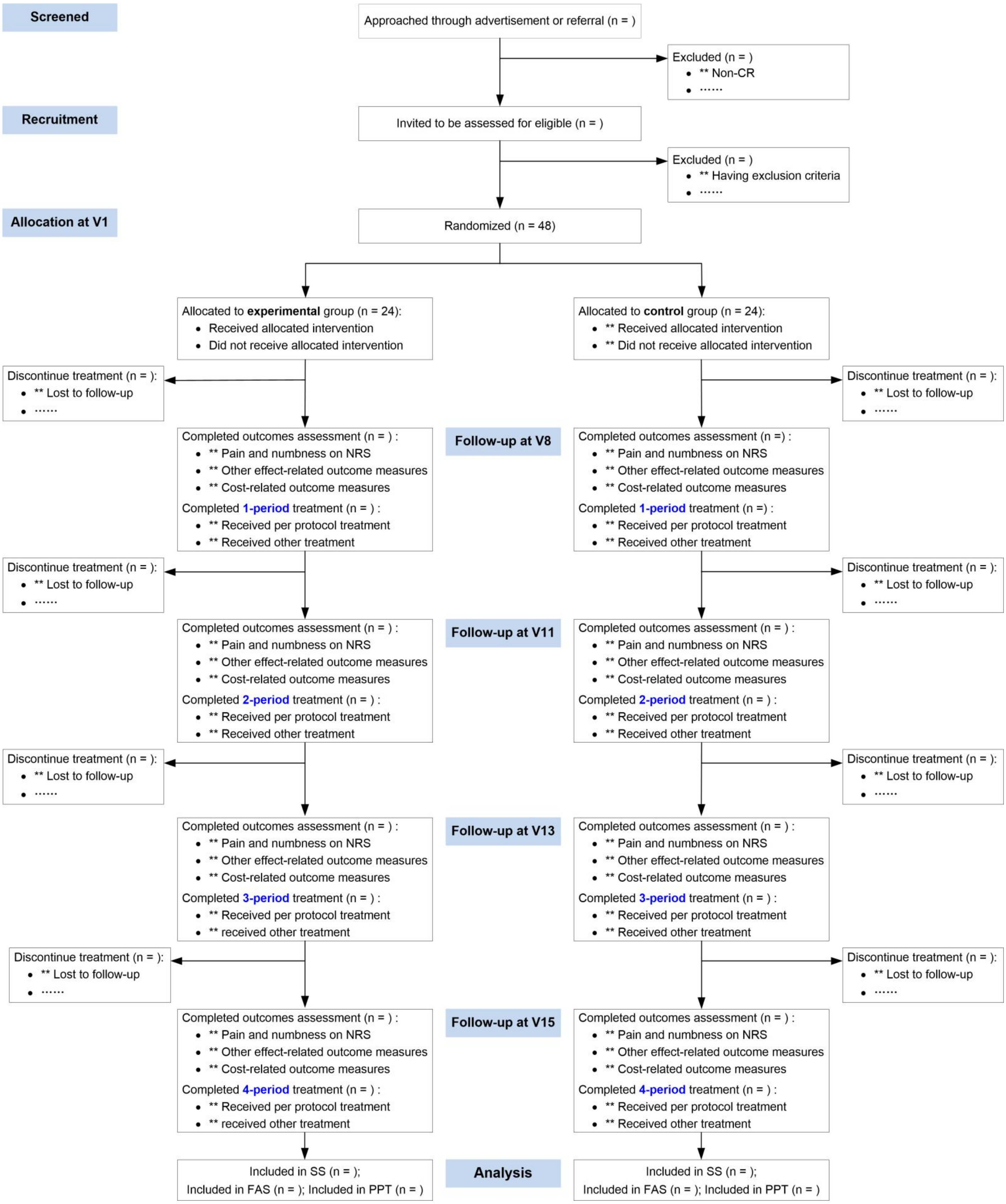

4 S4 Appendix. Ethics committee approval copy.

Initial Review: Meeting Review

Ethics Committe of Guangdong Provincial Hospital of Chinese Medicine

Notification of IEC Review Decision on *Pilot Study on Angled Manual Traction and Usual Care for Cervical Radiculopathy: a single-centre randomized controlled trial*

Application No. : BF2024-156-01

Review Date: May 31, 2024

Review Location: Room 2005-2006 (Oblong Conference Room), 20th Floor, Advanced Study Building, Guangdong Provincial Hospital of Chinese Medicine

Reviewed Project: Pilot Study on Angled Manual Traction and Usual Care for Cervical Radiculopathy: a single-centre randomized controlled trial

Reviewed Documents:
1. Initial review application form including financial disclosure statement
2. Approval document of funding
3. Study Assignment
4. Research protocol (version/date: 1.0 / 20240510)
5. Informed consent form (version/date: 1.0 / 20240510)
6. Recruitment materials for participants (including advertisements) (version/date: V1.0 / 20240510)
7. Clinical case report rorm (version/date: V1.0 / 20240510)
8. Participant diary (version/date: V1.0 / 20240510)
9. Resume of Principal investigator
10. GCP training certificate of Principal investigator
11. Research group list
12. Screening and enrollment form (version/date: V1.0/20240510)
13. Standard operating procedures (version/date: V1.0/20240510)

Sponsor / Task Issuing Unit: Guangdong Provincial Hospital of Chinese Medicine

Clinical Research Unit: Guangdong Provincial Hospital of Chinese Medicine (Department of Minimally Invasive Spine, Main Hospital)

Principal Investigator: Shaojun Liao

Participating Member: Lan Cheng, Xu-sheng Liu, Xiao-hui Qiu, Lin Wei, Li-chang Liu, Bo Liu, Li-kai Li, Qiu-yan Huang, Hao-xi Feng

Comments:

In compliance to the ethical principles set forth in the *Good Clinical Practice for Drugs*, *Good*

Clinical Practice for Medical Devices, and *Guiding Principles for Ethical Review of Drug Clinical Trials* by the State Food and Drug Administration; the *Measures for Ethical Review of Biomedical Research Involving Humans and Measures for the Administration of Clinical Research on Stem Cells (Trial)* by the National Health and Family Planning Commission; the *Standards for the Construction of Ethical Review Platforms for Clinical Research in Traditional Chinese Medicine* by the State Administration of Traditional Chinese Medicine; as well as the *Declaration of Helsinki* by the World Medical Association and the *International Ethical Guidelines for Health-related Research Involving Humans* by the Council for International Organizations of Medical Sciences, the Ethics Committee of Guangdong Provincial Hospital of Chinese Medicine has reviewed the project *Pilot Study on Angled Manual Traction and Usual Care for Cervical Radiculopathy: a single-centre randomized controlled trial* (Application No.: BF2024-156), and concludes: **Approved with Necessary Revisions**, with a follow-up review frequency (or adjusted) of 12 months.

The opinions are as follows:

I. Regarding the research protocol:
(1) It is recommended to exclude patients with osteoporosis;
(2) It is recommended to supplement the criteria for judging and contingency plans for managing cases where patients' symptoms cannot be relieved or even worsened.

II. Regarding the informed consent form:
In terms of costs, it is recommended to specify the specific types of "conventional treatment drugs".

Note: If protocol modifications involve content in other materials (such as the informed consent form, recruitment advertisements, case report forms, etc.), the other materials must also be updated accordingly.

Documents revised in accordance with the review opinions or statements of differing views on the review opinions shall be submitted as a "Re-review Application" to the Ethics Committee for review, and may be implemented only after approval. If you have any questions, please contact the Ethics Committee promptly.

Modifications to the protocol, informed consent form, or recruitment materials must include changes to the version number and version date, and the modified parts shall be marked with underlines. If the review opinion is "Approved with Revisions", please submit the re-review application within 1 month; if the review opinion is "Revised and Re-reviewed", please submit the re-review application within 3 months. Otherwise, it will be deemed an automatic waiver of the right to modify, and the study must be terminated or a new ethics review application must be submitted.

Contact Phone Number: Ethics Committee Office: 020-81887233-35943

Signature of Chariman : Lan Cheng
Date : June 13, 2024
Institutional Ethics Committee of Guangdong Provincial Hospital of Chinese Medicine
(Stamp)

Re-review: Expedited Review

Ethics Committee of Guangdong Provincial Hospital of Chinese Medicine Approval Notice

Approval No. BF2024-156-01

**Date of Review**	Initial Review: May 31, 2024; Re-review: June 13, 2024.
**Place of Review**	NO. 2005-2006 Conference Room, 20th Floor, Advanced Training Building, Guangdong Provincial Hospital of Chinese Medicine, No.111 Dade Road, Guangzhou, Guangdong Province, China
**Clinical Research Approval**	--
**Clinical Research Project**	Pilot Study on Feasibility of Angled Manual Traction for Cervical Radiculopathy: a single-center randomised controlled trial
**Reviewed Documents**	1. Re-review application form<br>2. Revised research protocol (version/date: 2.0 / 20240603)<br>3. Revised informed consent form (version/date: 2.0 / 20240603)<br>4. Initial review application form including financial disclosure statement<br>5. Approval document of funding<br>6. Study Assignment<br>7.Recruitment materials for participants (including advertisements) (version/date: V1.0 / 20240510)<br>8. Clinical case report rorm (version/date: V1.0 / 20240510)<br>9. Participant diary (version/date: V1.0 / 20240510)<br>10.Resume of Principal investigator<br>11. GCP training certificate of Principal investigator<br>12. Research group list<br>13. Screening and enrollment form (version/date: V1.0/20240510)<br>14. Standard operating procedures (version/date: V1.0/20240510)
**Sponsor**	Guangdong Provincial Hospital of Chinese Medicine
**Research Unit**	Department of Minimally Invasive Spine, Guangdong Provincial Hospital of Chinese Medicine
**Principal Investigator**	Shaojun Liao
**Review Mode**	Initial Review: Meeting Review; Re-review: Expedited Review.
**Participating Member**	Initial Review: Lan Cheng, Xu-sheng Liu, Xiao-hui Qiu, Li-chang Liu, Bo Liu, Li-kai Li, Qiu-yan Huang, Hao-xi Feng; Re-review: Xiao-hui Qiu, Li-chang Liu
**Comments**	In compliance to the ethical principles set forth in the "Good Clinical Practice for Drugs", "Good Clinical Practice for Medical Devices", and "Guiding Principles for Ethical Review of Drug Clinical Trials" by the State Food and Drug Administration; the "Measures for Ethical Review of Biomedical Research Involving Humans" and "Measures for the Administration of Clinical Research on Stem Cells (Trial)" by the National Health and Family Planning Commission; the "Standards for the Construction of Ethical Review Platforms for Clinical Research in Traditional Chinese Medicine" by the State Administration of Traditional Chinese Medicine; as well as the "Declaration of Helsinki" by the World Medical Association and the "International Ethical

	Guidelines for Health-related Research Involving Humans" by the Council for International Organizations of Medical Sciences, the Ethics Committee of Guangdong Provincial Hospital of Chinese Medicine agrees this clinical study design and perform the study with the reviewed documents.		
**Institutional Ethics Committee Statement**	This approval document will be conducted archival filing in other institutions of their ethics committee. Please contact us if there is any different opinion about the feasibility of this research (qualification/ experience of investigators, research facilities, etc.).<br>Please notify us when this research is suspended / terminated / completed. Severe adverse event or any unexpected adverse event might influence the balance of risk and benefit of the study, which should be reported to the ethics committee in time. Re-review is needed in case of protocol/inform consent form modification and principal investigator replacement, study begins after further approval. Affecting subject's willingness to participate in situations which is violate the program should be promptly reported. Please submit the study progress/final report a month before approval document expire date.<br>All research projects involving Chinese human genetic resources that require approval must obtain approval from the Human Genetic Resource Administration of China (HGRAC) before commencing research. Interventional clinical studies not conducted for product registration purposes must be registered in the National Medical Research Registration and Filing Information System (http://114.255.48.20) before initiation.		
**Term of Validity**	June 13, 2024 to June 13, 2026	**Periodicity of Follow-up**<br>**Date of Next Review**	1 year<br>June 13, 2025
**Contact Person**	Xiao-yan Li, Tel: 8620-81887233 ext35943		
**Signature of Chariman**	Lan Cheng		
	Institutional Ethics Committee of Guangdong Provincial Hospital of Chinese Medicine (Stamp)		
	Date: June 13, 2024		
